# Relationships between the clinical characteristics and copy numbers of DNA of cytomegalovirus determined by real-time PCR

**DOI:** 10.1007/s10792-020-01412-6

**Published:** 2020-05-16

**Authors:** Ayano Yoshimura, Kaoru Araki-Sasaki, Noriko Toyokawa, Rho Fujiwara, Nobuo Jho, Fumi Gomi

**Affiliations:** 1 Japan Health Care Organization, Hoshigaoka Medical Center, Hirakata, Japan; 2 Department of Ophthalmology, Hyogo College of Medicine Hospital, Nishinomiya, Japan; 3 Nagata Eye Clinic, Nara, Japan; 4 Department of Ophthalmology, Kansai Medical University, 2-5-1 Shinmachi, Hirakata City, Osaka 5731010 Japan; 5 Jho Eye Clinic, Hirakata, Japan

**Keywords:** Keratic precipitates, Corneal endotheliitis, Cytomegalovirus, Coin-shaped lesion, Sectoral corneal edema

## Abstract

**Purpose:**

To determine whether there is a correlation between the clinicals characteristics including various types of keratic precipitates and the copy numbers of the DNA of cytomegalovirus (CMV) in eyes with CMV corneal endotheliitis.

**Methods:**

We reviewed the medical charts of four cases of corneal endotheliitis that were CMV-positive. We have classified types of clinical phenomenon into four types: coin-shaped KPs, sectoral corneal edema with or without Khodadoust line-like KPs, mutton-fat KPs, and fine KPs and have graded their severity. We also determined the copy numbers of the DNA of CMV in the aqueous humor by real-time polymerase chain reaction before and during the treatment. We evaluated the correlation between the patterns of clinical characteristics and copy number of the DNA of CMV.

**Results:**

There were clinical improvements in all eyes following topical ganciclovir in conjunction with low dose of topical steroid treatment, with or without oral valganciclovir. The clinical characteristics and the copy numbers of the DNA of CMV varied during the treatment period. The presence of coin-shaped KPs was correlated with high copy numbers (10^5^–10^3^ copies/ml) of the DNA of CMV. The copy numbers of the DNA of CMV with sectoral corneal edema with or without Khodadoust line-like KPs ranged from 10^4^ to 10^2^ copies/ml, and it was occasionally accompanied by high intraocular pressure. Mutton-fat KPs were observed inferiorly, sometimes together with coin-shaped KPs and sectoral corneal edema, or solely. The copy numbers in eyes with mutton-fat KPs varied and occasionally less than the cutoff level. Fine-pigmented KPs were observed after the resolution of the endotheliitis, and no DNA of CMV was detected in the aqueous humor.

**Conclusions:**

Careful observations of the clinical characteristics such as the KPs and corneal edema might be helpful in estimating the amount of the DNA of CMV in eyes with corneal endotheliitis.

## Introduction

Corneal endotheliitis, first characterized by Khodadoust and Attarzadeh [1], is characterized by the presence of linear keratic precipitates (KPs) similar to that found in eyes with corneal endothelial rejection. Later, Ohashi et al. [2] reported on a unique corneal endothelialitis that consisted of severe stromal edema associated with keratic precipitates. This endotheliitis was of viral origin, especially the herpes simplex virus (HSV). Additionally, Amano [3] reported that the herpes simplex virus can cause trabeculitis and increased intraocular pressure (IOP) in patients with corneal endotheliitis. In 2006, Koizumi et al. [4] identified cytomegalovirus (CMV) as one of the causative agents of corneal endotheliitis and this was confirmed by Chee et al. [5]. Subsequently, the characteristics of this clinical entity, such as coin-shaped regions and Khodadoust line-like KPs with corneal edema, were reported in eyes with corneal endotheliitis by a multicenter clinical study [6].

CMV endotheliitis is associated with a marked decrease in corneal endothelial cell densities even with mild anterior chamber inflammation. This corneal endothelial cell damage is associated with recurrent inflammations, IOP elevations, and mostly high CMV viral loads [7, 8]. To prevent the visual loss by corneal decompensation in CMV endotheliitis, an appropriate strategy for the treatment based on the DNA viral load is necessary. However, viral infection can sometimes evoke strong immune reactions even with slight amount of virus. To the contrary, anterior chamber-associated immune deviation (ACAID) can mask the clinical phenomenon of the endotheliitis [9, 10]. Therefore, the clinical severity of the anterior chamber inflammation does not always reflect the amount of virus DNA and it is very difficult to predict the amount of virus only from the severity of inflammation such as injection and number of cells in the aqueous humor.

From our clinical experience with many cases of CMV-associated corneal endotheliitis, we noted that the type of KPs and corneal edema could be a marker of the morbidity of the anterior segment inflammation caused by CMV.

Thus, the purpose of this study was to determine whether there is a correlation between various clinical characteristics and the copy number of the DNA of CMV in the aqueous humor of four patients with CMV-proven corneal endotheliitis.

## Methods

### Subjects

The medical information of four patients who were diagnosed with CMV endotheliitis was collected from the Japan Community Health Care Organization (JCHO), the Hoshigaoka Medical Center, and the Nagata Eye Clinic between September 2016 and July 2019. All of the cases were treated as Posner-Schlossman syndrome or unknown iridocyclitis with low dose of steroid.

The study procedures were approved by the Ethics Committee of the JCHO, the Hoshigaoka Medical Center, and Nagata Eye Clinic, and the procedures conformed to the tenets of the Declaration of Helsinki. The written informed conset was obtained from the patients for ths study.

The demographics and clinical findings including the age, sex, duration of the disease, best-corrected visual acuity (BCVA), and IOP of each patient were recorded. Photographs of the KPs and sectoral corneal edema were obtained by slit-lamp biomicroscopy, and the clinical characteristics were determined by two or more authors from an examination of the photographs. The corneal endothelial cell density was determined with a noncontact-type specular microscope (Tomey EM-3000, Nagoya, Japan). Topical 0.5% ganciclovir (GCV) was prepared in the hospital dispensaries from vials designed for intravenous infusion and was applied to all of the patients after the diagnosis of CMV endotheliitis. A signed informed written consent was obtained from all the patients for the use of the GCV eye drops and the sampling of the aqueous humor. The CMV DNA copy numbers were determined by real-time qualitative PCR as described in detail elsewhere [11].

### Classification of patterns of clinical characteristics and grading of severity of endotheliitis

We focused on four clinical characteristics: coin-shaped, circularly arranged KPs, sectoral corneal edema with/without linear KPs resembling Khodadoust line similar to those observed in corneal endothelial rejection, mutton-fat KPs over the inferior cornea, and fine-white or -pigmented KPs. The severity of each characteristics was ranked into three grades; high (++), intermediate (+), and low to none (−) by two experienced ophthalmologists listed in Table 1.

**Table 1:** Patterns of clinical characteristics with different types of KPs and Grading of their severity observed under the slit-lamp examination

KPs forms	Severity		
	High (++)	Intermediate (+)	None (−)
Coin-shaped KPs	More than three	One or two	None
Sectoral corneal edema with/without Khodadoust line-like KPs	Visible by diffuse illumination	Visible by slit beam	None
Mutton-fat KPs	More than ten	Less than ten	None
Fine KPs (pigmented or white)		Recognized	None

KPs: keratic precipitates

## Results

### Subjects

**Fig. 1:**
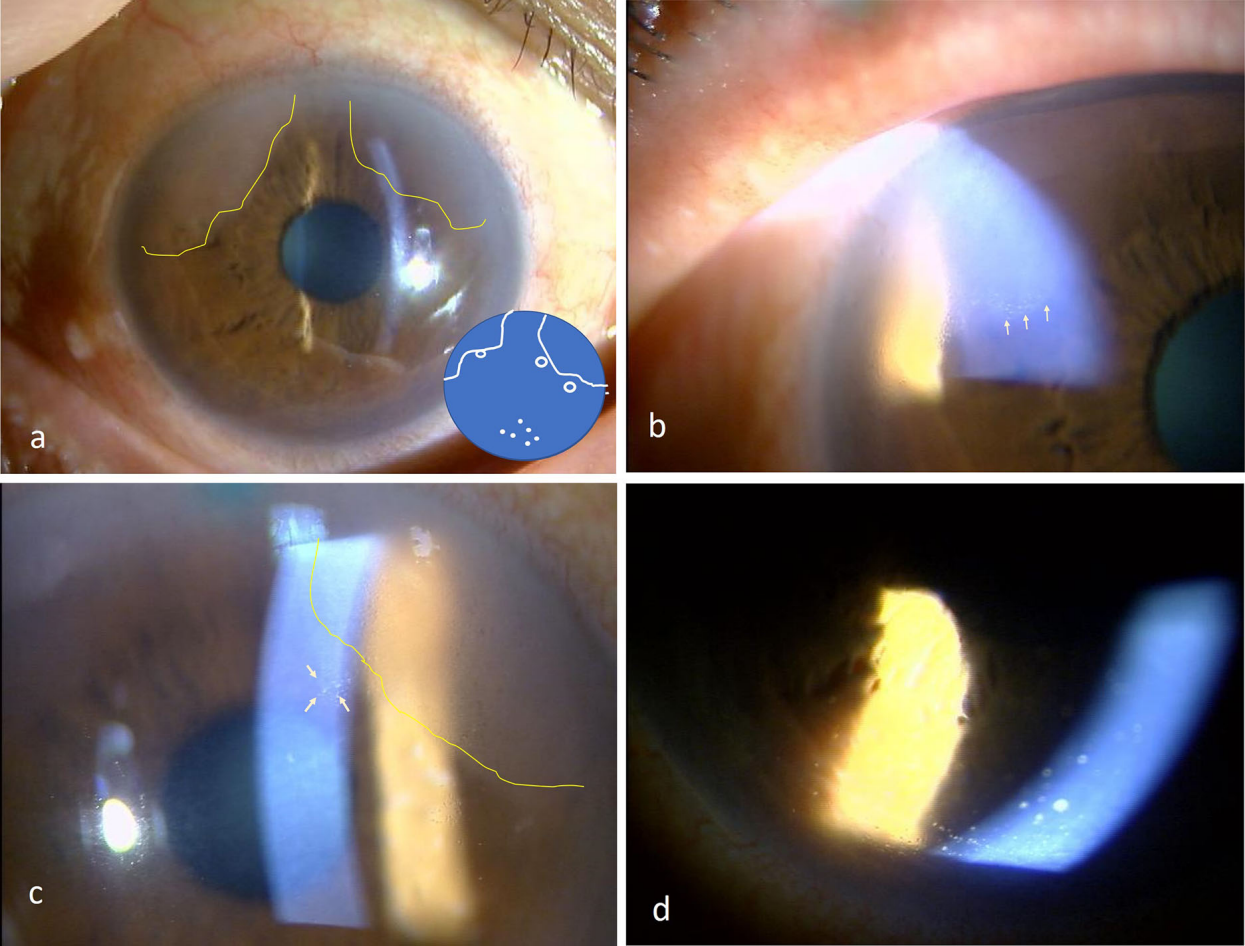
Slit-lamp photographs of Case 1 at the time of diagnosis. **a** Sectoral corneal edema can be seen at 2 o’clock and 11 o’clock. Inset: Schematic image of (**a**–**d**). The amount of the DNA of cytomegalovirus (CMV) was 9.4 × 10^4^ copies/ml. **b** Khodadoust line-like KPs (arrows) are present at the leading edge of the edema. **c** Magnified image at 2 o’clock. The coin-shaped KPs (arrows) can be seen ahead of the Khodadoust line-like lesion (arrowheads). **d** Magnified image at 6 o’clock showing mutton-fat KPs

**Fig. 2:**
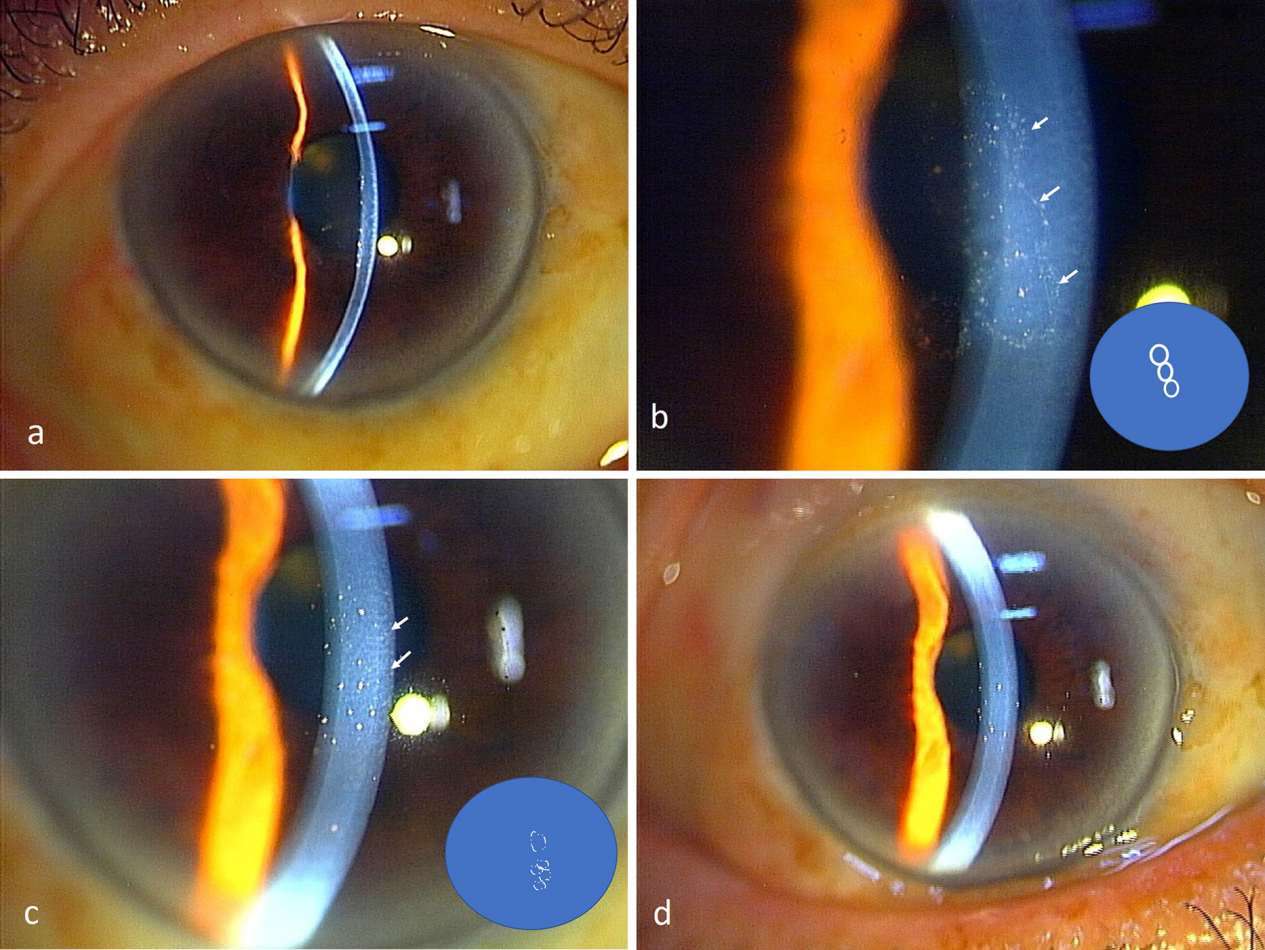
Slit-lamp photographs of the cornea of Case 2. **a** At the time of the diagnosis, three coin-shaped KPs can be seen in the center of the cornea. The inflammation of the anterior chamber is mild. **b** Magnified image of the three coin-shaped KPs (arrows). Khodadoust line-like KPs and mutton-fat KPs are not present. The copy number of CMV DNA was 2.8 × 10^4^ copies/ml. Inlet: schematic image of (**b**). **c** Photograph 2 weeks after the beginning the treatment. The three coin-shaped KPs are slightly reduced in size (arrows) but are still present. The copy numbers of the DNA of CMV in the aqueous humor was still 1.3 × 10^5^ copies/ml. Inset: schematic image of (**c**). **d** Two months later. Only pigmented KPs are observed. The copy numbers of CMV DNA in the aqueous humor were less than the detection level

**Fig. 3:**
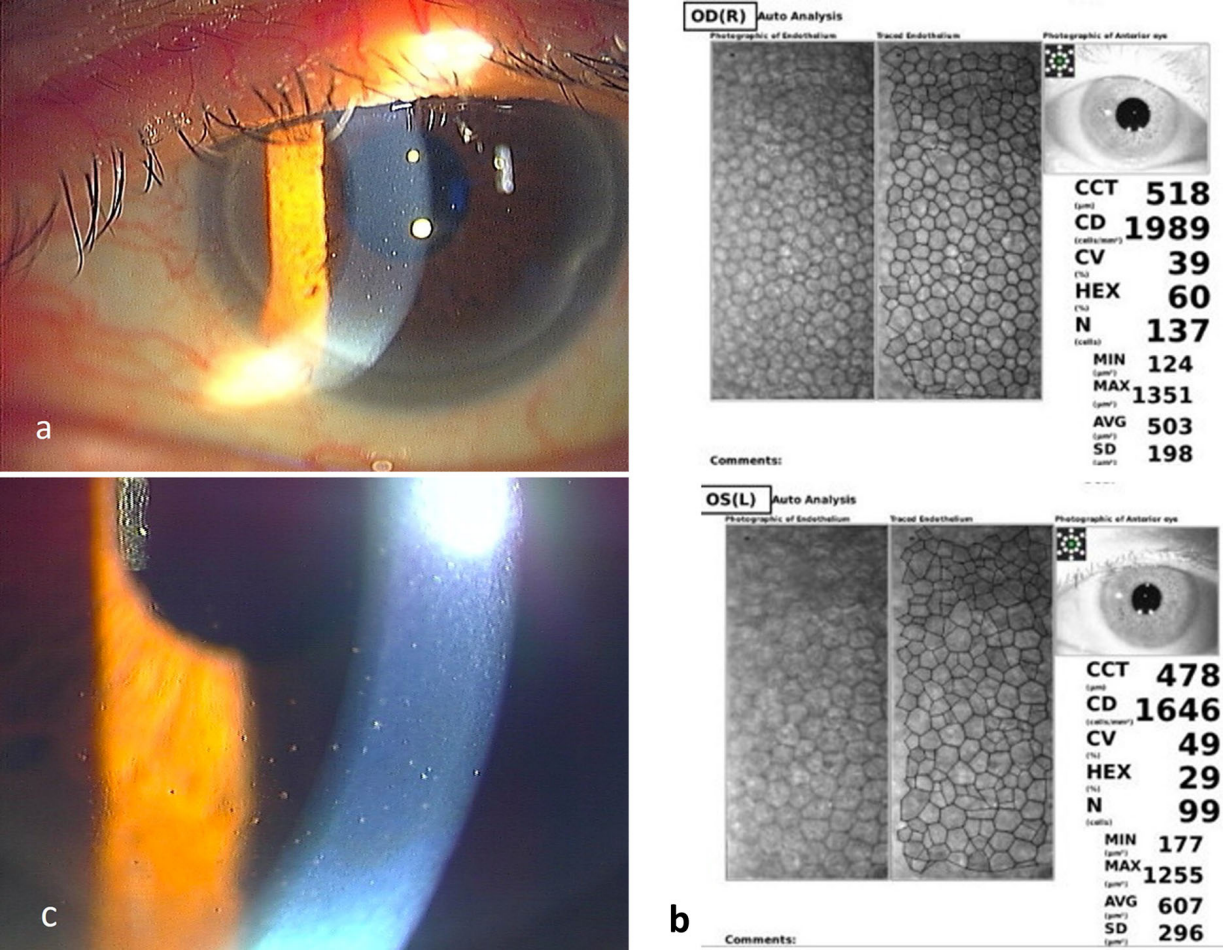
Slit-lamp photographs and specular image of Case 3. **a** Only white fine KPs can be seen at the time of diagnosis. PCR of the aqueous humor showed 9.4 × 10^1^ copies/ml of the DNA of CMV. **b** Specular microscopic image shows the difference in the number of endothelial cells between the two eyes. The number of endothelial cells is reduced only in the affected left eye. This specular image led us to suspect CMV endotheliitis. **c** One year after the treatment, slight inflammation with small mutton-fat KPs can be seen. However, no virus DNA was detected in the aqueous humor. Finally, these KPs turned to the pigmented KPs, and CMV was not detected by PCR

**Fig. 4:**
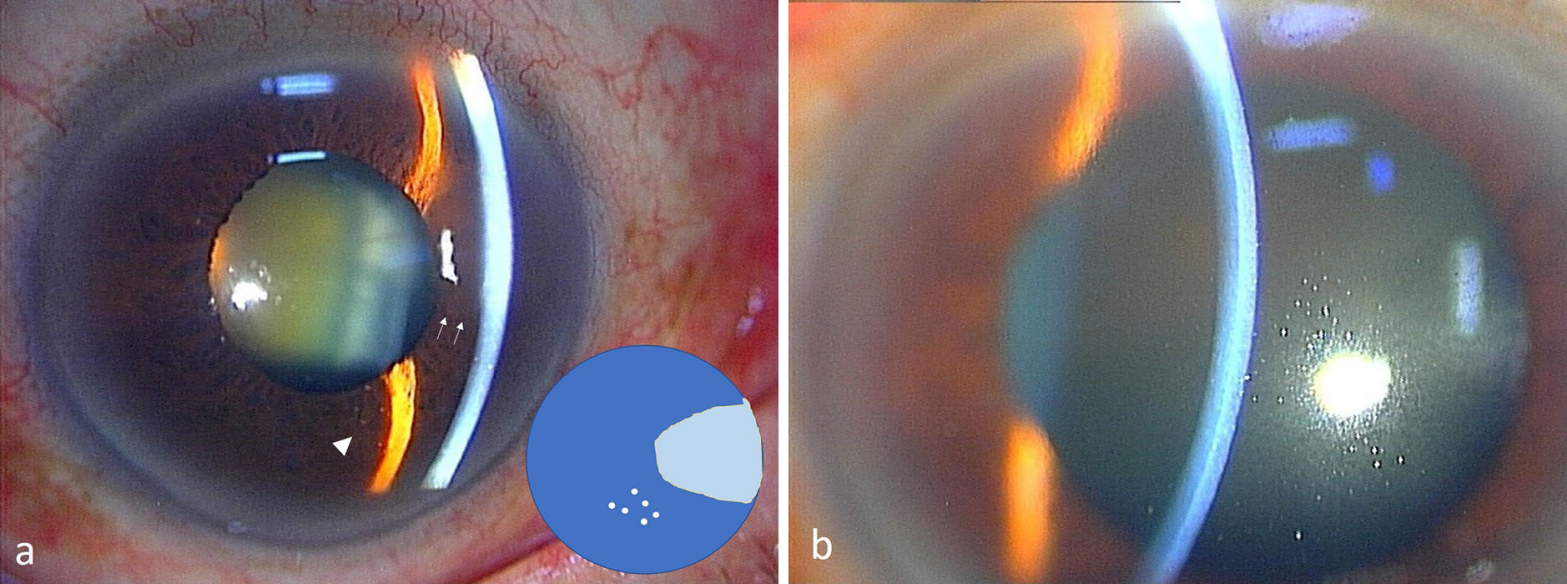
Slit-lamp photographs of Case 4. **a** Triangular corneal edema surrounded by slight Khodadoust line-like KPs (arrows) can be seen at 3 o’clock. Although mutton-fat KPs (arrowheads) are obvious at the inferior part of the cornea, coin-shaped KPs are absent. The amount of CMV DNA was 5.0 × 10^2^ copies/ml. Inlet: Schematic image of (**a**). **b** Partial corneal edema with mutton-fat KPs is present even with the continuous use of steroid and GCV eye drops. The intraocular pressure gradually increased and reached 50 mmHg

All four patients were immunocompetent Japanese men, 72–76 years of age (average: 74.8 years), and the diagnosis of CMV endotheliitis was confirmed by qualitative RT-PCR examination of the aqueous humor. All cases were positive for CMV and negative for HSV and VZV. None of the patients had a history of any systemic diseases. Serology examinations for human immunodeficiency virus were negative. All cases were initially diagnosed as Posner-Schlossman syndrome or unilateral anterior uveitis of unknown origin with ocular hypertension. They were treated with corticosteroid eye drops for more than one year by local ophthalmologists before they were referred to us. The photographs obtained by slit-lamp examinations at the time of diagnosis of CMV are shown in Figs. 1, 2, 3, and 4. All four patients were treated with topical 0.5% GCV, and two patients also received oral valganciclovir for 14 days. Topical steroid such as 0.1% fluorometholone was applied continuously before and after the diagnosis of CMV endotheliitis. In Case 4, fluorometholone was switched to 0.1% dexamethasone at the time of severe inflammation with IOP elevation. Topical glaucoma medications were used on all four eyes before and after the diagnosis. Oral acetazolamide tablets were used concurrently in one of four eyes. The IOPs were controlled with medications except in Case 4 which had a recurrent sectoral corneal edema with slight Khodadoust line-like KPs. Clinical improvements and lower CMV copy numbers in the aqueous humor were confirmed following anti-viral treatments in conjunction with the steroid eye drops.

### Clinical manifestations

The photographs of the anterior segment at the time of diagnosis of CMV endotheliitis are shown in Figs. 1, 2, 3 and 4.

In Case 1, various patterns of characteristics, e.g., coin-shaped KPs, sectoral corneal edema with Khodadoust line-like KPs, and mutton-fat KPs over the inferior cornea, were observed before and during the course of treatment. The corneal edema extended from the limbus to the Khodadoust line-like KPs at 2 and 11 o’clock (Fig. 1a, b). Some of the coin-shaped KPs (arrows) were observed at the para-central zone of the cornea adjacent to the sectoral corneal edema demarcated by Khodadoust line-like KPs (arrowheads Fig. 1c). Mutton-fat KPs were detected at the inferior zone of the cornea (Fig. 1d). The amount of CMV DNA in the aqueous humor was 9.4 × 10^4^ copies/ml before the application of GCV. These KPs were gradually resolved after beginning of the treatment, and they turned to fine-pigmented KPs at 6 months after start of the treatment. No CMV DNA was detected at this time by RT-PCR.

In Case 2, three coin-shaped KPs were detected at the center of the cornea at the initial examination (Fig. 2a, b). Sectoral corneal edema, Khodadoust line-like KPs, and mutton-fat KPs were not observed. The conjunctival injection was mild, and there were few inflammatory cells in the aqueous humor. The copy number of the DNA of CMV in the aqueous humor before the application of GCV was 2.8 × 10^4^ copies/ml. At 2 weeks after the start of the topical GCV, the coin-shaped KPs were still present although there was a slight decrease in the size (Fig. 2c). The viral copy number was 1.3 × 10 copies/ml. With continued treatment, the coin-shaped KPs gradually turned to the fine-pigmented KPs in two months (Fig. 2d). At this time, the DNA of CMV was not detected in the aqueous humor by RT-PCR.

In Case 3, only white fine KPs were recognized at the time of diagnosis (Fig. 3a), but there was a clinical history of decreased endothelial cell density with long duration uveitis in his left eye (Fig. 3b). These findings led us to suspect CMV endotheliitis, and the examination of the aqueous humor by RT-PCR revealed 9.4 × 10 copies/ml of the DNA of CMV. With the application of topical GCV and steroids for one month, the inflammation was resolved with no recurrences. Although mutton-fat KPs appeared transiently at the inferior part of the cornea at one year after the treatment (Fig. 3c), the copy numbers of CMV DNA were less that the detection level and the inflammation resolved spontaneously.

In Case 4, the initial examination showed that triangular-shaped sectoral corneal edema with slight amount of Khodadoust line-like KPs was observed at 3 o’clock, with mild inflammation in the anterior chamber (Fig. 4a). This was accompanied by mutton-fat KPs over the inferior part of the cornea. The amount of CMV DNA in the aqueous humor was 5.0 × 10^2^ copies/ml at this time. The anti-viral treatment led to a gradual disappearance of the KPs, and the cornea edema was resolved. However, three months later, the sectoral corneal triangle edema demarcated by Khodadoust line-like KPs with ciliary injection and severe inflammatory cells in the aqueous humor were recognized even under the continuous use of GCV eye drops and reinforcement with steroids. The IOP gradually increased and reached 50 mmHg (Fig. 4b). The marked IOP elevation was uncontrollable even with maximum anti-glaucoma medications including oral acetazolamide, and a trabeculectomy was performed. A tap of the aqueous humor to detect CMV DNA was not performed at that time because we could not obtain the patient’s consent. After the surgical treatment, the edema and inflammation were resolved and KPs were turned to pigmented cells with good IOP control and no virus was detected by RT-PCR.

### Relationship between the clinical characteristics and copy numbers of DNA of CMV

The appearances of clinical characteristics were carefully documented at each observation point, and the anterior chamber taps were performed at the time of the clinical examinations and used for the real-time PCR. The patterns of the clinical characteristics and CMV DNA copy numbers are presented in Table 2. At the initiation of anti-viral therapy, coin-shaped KPs were detected in two eyes (Cases 1 and 2), sectoral corneal edema with Khodadoust line-like KPs in two eyes (Cases 1 and 4). The presence of the coin-shaped KPs was always associated with high copy numbers of CMV DNA of ≥ 10^3^ copies/ml in the aqueous humor. In the eyes with sectoral corneal edema with Khodadoust line-like KPs, the copy numbers of CMV DNA varied from 5.0 × 10^2^ copies/ml to 9.4 × 10^4^ copies/ml. The coin-shaped KPs in Cases 1 and 2 resolved after a 4-week treatment period and fine KPs appeared in response to the GCV treatment. The sectoral corneal edema with Khodadoust line-like KPs was also resolved after topical GCV in Cases 1 and 4; however, it recurred in Case 4 which had a marked elevation of the IOP to nearly 50 mmHg and required a trabeculectomy. The mutton-fat KPs were usually accompanied by coin-shaped KPs or sectoral corneal edema with or without Khodadoust line-like KPs, but not always. In Case 3, mutton-fat KPs were observed solely after the treatment for one year with topical corticosteroid and GCV. However, the viral copy number was less than the detection level in the aqueous humor. The fine-white and -pigmented KPs appeared after the resolution of corneal endotheliitis in all cases, and they were associated with the low or no copy numbers of CMV (DNA of ≤ 10 copies/ml).

**Table 2:** Details of clinical characteristics with different types of KPs forms and the CMV DNA copy numbers in the humor determined by PCR

CMV copy number (10^n^)	5	4		3	2	1	Less than the cutoff level			
Coin-shaped lesion	++	++	++	+	−	−	−	−	−	−
Sectoral corneal edema with/without Kodadoust-like line	−	++	−	+	++	−	−	−	−	−
Mutton-fat KPs	−	++	−	+	++	−	−	−	+	−
Fine KPs, white	−	−	−	−	−	+	−	−	+	−
Fine KPs, pigmented	−	−	−	−	−	−	+	+	−	
Case number	2	1	2	1	3	4	1	2	3	4

## Discussion

There are two major difficulties that need to be considered when treating CMV endotheliitis. First, there is no definitive evidence on the need of steroids along with anti-viral drugs for a successful outcome. Second, there are no experimentally derived criteria for when to terminate the GCV treatment. There are some concerns that immunosuppressive agent such as steroids might evoke a viral re-activation because this disease is well known to have high recurrence rates [12, 13]. Both of these difficulties are due to the inability to determine the activity of CMV activity or virus titers from the clinical manifestations.

We have classified the clinical characteristics with various kinds of KPs into four types based on Moshirfar’s review [14]. We found that the clinical characteristics were related to the copy number of DNA of CMV in the aqueous humor.

Kandori et al. [7] and Miyanaga et al. [8] had already reported on the relationship between the type of KPs and viral level. However, they examined only one time point in each case and did not describe the changes during the course of the disease process. We have followed the changes of the DNA amounts along with the treatment process and found new findings on the relationships between the clinical characteristics and the copy numbers of the DNA of CMV. First, the eyes with coin-shaped KPs had copy numbers of the DNA of CMV of ≥ 10^3^ copies/ml in the aqueous humor. This observation is supported by Kandori’s report [7]. Shiraishi [15] also reported that the edema with the coin-shaped KPs was observed as “owl’s eye” lesions by specular microscopy which is similar to the virus plaque of cells in vitro. Taken together with our findings, the coin-shaped lesions must be the site where the endothelial cells are infected with high titers of CMV. This characteristic appearance indicates the need for rigorous anti-viral treatment.

Second, the copy numbers of CMV DNA in eyes with sectoral corneal edema with/without the Khodadoust line-like KPs ranged from 5.0 × 10^2^ to 9.4 × 10^4^ copies/ml. Khodadoust line-like KPs were not always accompanied by high copy numbers of the DNA of CMV. As was reported in HSV endotheliitis [3], experimental research has shown that CMV infects the cells of the trabecular meshwork [16, 17]. And sectoral corneal triangular edema with or without Khodadoust line-like KPs usually appears connected to the limbus. Thus, we suggest that sectoral corneal edema is occasionally accompanied by Khodadoust line-like KPs which might be a reactive phenomenon due to active CMV infection in the trabecular meshwork tissue. Khodadoust line-like KPs are usually accompanied by the sectoral peripheral corneal edema [6] which can sometimes be difficult to observe because of the edematous opacity. The variations in the severity of Khodadoust line-like KPs on sectoral corneal edema might be correlated with a range of copy numbers of the DNA of CMV.

Third, the mutton-fat KPs in the inferior part of the cornea are usually observed in various types of anterior uveitis as immune reactions regardless of the type or titer of the organisms. From all of these observations, sectoral corneal edema with and without Khodadoust line-like KPs and mutton-fat KPs might indicate the necessity of the steroid therapy along with the anti-virus therapy.

Fourth, the eyes with fine KPs had low or no copy number of CMV DNA. The fine-pigmented KPs were specifically observed after the resolution of corneal endotheliitis in all of the eyes. Thus, the fine KPs might be a marker to terminate the anti-viral drug therapy.

Several clinical signs are usually seen concomitantly, and the most severe conditions affect the copy numbers of the DNA of CMV. For example, if coin-shaped KPs were observed with sectoral edema with Khodadoust line-like KPs or mutton-fat KPs, the coin-shaped KPs would be the most affected characteristic to determine the DNA amount.

Previously, Miyanaga et al. [8] reported that there was no significant correlation between the viral load and type of KPs. They studied seven cases of iridocyclitis and four cases of endotheliitis and observed only mutton-fat KPs and fine KPs. They focused on the association of viral loads and endothelial cell loss. They did not examine the relationships between the types of KPs and CMV copy numbers. Therefore, our observations are not in conflict with their observations.

Glucocorticoid resistance was recently reported in cases of CMV ulcerative colitis because of receptor alterations [18]. Cases 3 and 4 had recurrences of the anterior chamber inflammation while being treated with topical steroids and GCV. These recurrences might be related to the resistance against steroid used for the CMV infection. Further studies on the viral load may be helpful in clarifying the resistance of CMV to steroid therapy and IOP elevation.

This study has some limitations including its retrospective nature. All cases had been treated with steroid eye drop for iridocyclitis before they were referred to us. The use of steroids might have affected the copy numbers. The use of anti-viral drug also might have affected the results. In addition, the timing of sample collection was not standardized in each patient. Another limitation is the small sample size of four patients. However, the speculation of the copy numbers of CMV-DNA during the course of anti-viral treatment is important in clinical practice. Thus, we believe that the results of current study would propose some information to speculate the copy numbers of CMV-DNA in the treatment of CMV corneal endotheliitis.

In conclusion, the clinical characteristics and different types of KPs and edemas with their chronological changes might be important signs of the viral load in eyes with CMV corneal endotheliitis. In the era of the development of ganciclovir gel for the treatment of anti-CMV endotheliitis [19], further large-scale studies are needed to conclude the contribution of various characteristics with different KPs in the management of CMV endotheliitis.
